# Inferior vena cava filters in pregnancy: Safe or sorry?

**DOI:** 10.3389/fcvm.2022.1026002

**Published:** 2022-11-07

**Authors:** Ingrid M. Bistervels, Andrea Buchmüller, Bernard Tardy

**Affiliations:** ^1^Department of Vascular Medicine, Amsterdam UMC Location University of Amsterdam, Amsterdam, Netherlands; ^2^Amsterdam Cardiovascular Sciences, Pulmonary Hypertension and Thrombosis, Amsterdam, Netherlands; ^3^INSERM, Clinical Investigator Center 1408 – Centre Hospitalier Universitaire de Saint-Etienne, Saint-Etienne, France; ^4^F CRIN, INNOVTE Network, Saint-Etienne, France

**Keywords:** venous thromboembolism, pregnancy, safety, anticoagulants, vena cava filter

## Abstract

**Background:**

Potential hazards of vena cava filters include migration, tilt, perforation, fracture, and in-filter thrombosis. Due to physiological changes during pregnancy, the incidence of these complications might be different in pregnant women.

**Aim:**

To evaluate the use and safety of inferior vena cava filters in both women who had an inferior vena cava filter inserted during pregnancy, and in women who became pregnant with an inferior vena cava filter *in situ*.

**Methods:**

We performed two searches in the literature using the keywords “vena cava filter”, “pregnancy” and “obstetrics”.

**Results:**

The literature search on women who had a filter inserted during pregnancy yielded 11 articles compiling data on 199 women. At least one filter complication was reported in 33/177 (19%) women and included in-filter thrombosis (*n* = 14), tilt (*n* = 6), migration (*n* = 5), perforation (*n* = 2), fracture (*n* = 3), misplacement (*n* = 1), air embolism (*n* = 1) and allergic reaction (*n* = 1). Two (1%) filter complications led to maternal deaths, of which at least one was directly associated with a filter insertion. Filter retrieval failed in 9/149 (6%) women. The search on women who became pregnant with a filter *in situ* resulted in data on 21 pregnancies in 14 women, of which one (6%) was complicated by uterine trauma, intraperitoneal hemorrhage and fetal death caused by perforation of the inferior vena cava filter.

**Conclusion:**

The risks of filter complications in pregnancy are comparable to the nonpregnant population, but could lead to fetal or maternal death. Therefore, only in limited situations such as extensive thrombosis with a contraindication for anticoagulants, inferior vena filters should be considered in pregnant women.

## Introduction

Vena cava filters are intravascular devices that trap thrombi migrating from deep veins toward the pulmonary arteries, and therefore prevent new pulmonary embolisms. Currently, major guidelines agree on the recommended use of vena cava filters in patients with acute venous thromboembolism (VTE, comprising deep vein thrombosis [DVT] and pulmonary embolism) while therapeutic anticoagulant treatment is contraindicated if there is active bleeding or a high risk of bleeding—such as recent or planned surgery or delivery, and in patients with recurrent VTE despite adequate anticoagulant treatment (1–5). Complications occurring directly after insertion of the vena cava filter include access site thrombosis, infection, bleeding and perforation of the vena cava wall (2, 4, 6). Long-term complications of vena cava filters can occur in the days or months after insertion and include filter migration, filter tilt, perforation of the vena cava wall, fracture and embolization of filter struts, or in-filter thrombosis with or without concomitant deep-vein thrombosis (2, 6). These complications have been reported in 7–22% of the nonpregnant population (7, 8). Failure of filter retrieval was reported in 11–12% of nonpregnant patients (8, 9).

When a VTE occurs during pregnancy, the indicated anticoagulant treatment should temporarily be interrupted around time of delivery. This poses hemostatic challenges when VTE is diagnosed shortly prior to the expected date of delivery, since the risk of progression or recurrence of VTE is highest during the first month after diagnosis, while at the same time anticoagulant treatment can worsen peripartum bleeding.

Due to physiological changes that occur during pregnancy, pregnant women may be at increased risk of inferior vena cava filter complications. As a result of the dilated and curved inferior vena cava during pregnancy, the filter might be more likely to tilt and/or migrate, which could make the filter less effective and harder to retrieve. Moreover, the effect of compression of the gravid uterus on the inferior vena cava, contractions and increased intra-abdominal pressure while pushing, has not yet been established. Therefore, evidence-based guidance on the use of vena cava filters in pregnant women is paramount. In this review we aim to provide an overview of the available literature on the use and safety of inferior vena cava filters in pregnant women. We will separately report results for women who got an inferior vena cava filter inserted during pregnancy and for women who became pregnant with an inferior vena cava filter *in situ*.

## Inferior vena cava filters for acute venous thromboembolism inserted during pregnancy

In the first part of this review, we aim to evaluate the use, obstetric outcomes, and filter complications of patients who had an inferior vena cava filter inserted during pregnancy.

## Literature search—methods

A systematic search of literature published between January 2015 and May 2022 was conducted on Medline and Embase. The search strategy was based on the following keywords: “vena cava filter”, “pregnancy” and “obstetrics”. We searched for original studies, case series and case reports. No restrictions with regard to study design or geographic location were applied. Articles were included if they reported data on inferior vena cava filters inserted during pregnancy. Information about filter indication, route and timing of filter insertion, filter complications, indwelling time, and maternal and fetal outcomes was collected. All reference lists of included manuscripts were manually searched to identify related articles that were not yet identified.

## Results

**Figure 1:**
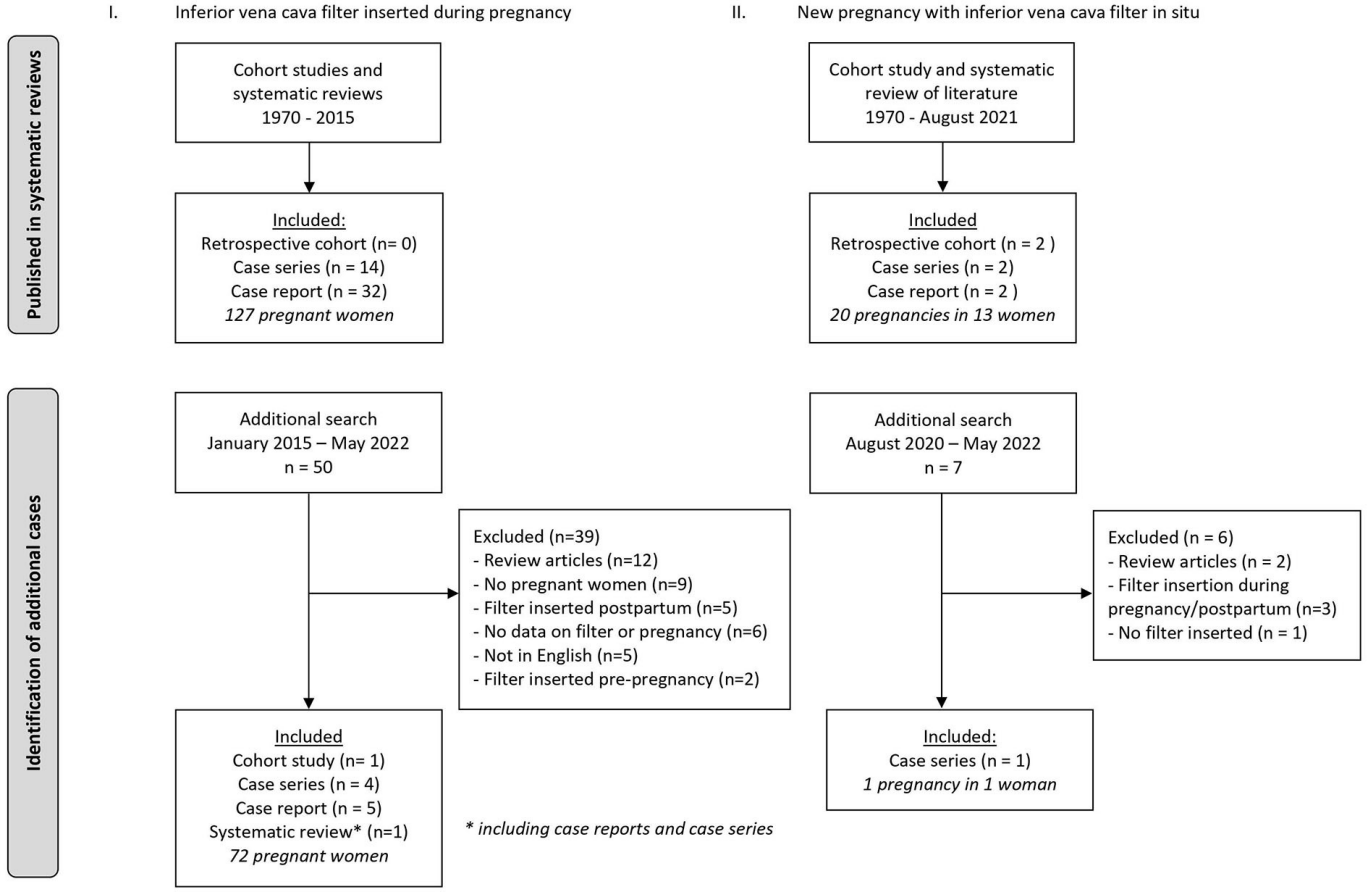
Flow chart literature searches.

Literature search yielded 50 articles based on titles and abstracts, and eleven articles were included after full text screening: one cohort study (10), four case series (8, 11–13), five case reports (14–18) and a systematic review with case series and case reports (19). The reasons for excluding the other 39 manuscripts were: review articles without case reports (*n* = 12), not concerning pregnant women (9), postpartum filter insertion (*n* = 5), article not in the English language (*n* = 5), no details provided concerning either the pregnancy or the filter (*n* = 6), and filter insertion prior to pregnancy (*n* = 2) (Figure 1). One of the included articles was a case report accompanied by an overview of the English language literature from January 1970 to 2014 on vena cava filters during pregnancy (14). In this overview (14), a total of 64 cases were reported and all these cases—except for three—were also included in another systematic review on inferior vena cava filters in pregnancy, published in 2016 (19). In a case series published in 2015 (11), 11 of the 20 cases were duplicates of previously published cases (20) included in the systematic review of Harris et al (19). From this article (11) we only retained the nine cases which were never previously published. Hence our systematic search yielded a total of 199 women who had an inferior vena cava filter inserted during pregnancy.

### Filter insertion

Of the 199 pregnant women, 45 women (23%) had a permanent filter (36 Greenfield, 4 Cardial, 2 Bird's Nest, 1 TrapEase, 2 undetermined) and 154 women (77%) had a retrievable filter (26 Neuhaus Protect, 20 Günther Tulip, 19 OptEase, 12 Antheor, 10 ALN, 5 Tempofilter, 4 Celect, 2 Recovery, 1 Zontik, 1 Prolyser, 1 Cardial and 53 undetermined) inserted. The filter locations were reported for 138 women: inferior vena cava filters were inserted in a suprarenal position in 96 women (70%) and in an infrarenal position in 42 women (30%).

The indication for filter insertion in all women was venous thromboembolism during pregnancy: 90 women (45%) had a proximal DVT, 17 women (9%) had pulmonary embolism with or without concomitant DVT, and in 51 women (26%) the exact thrombosis location remained unspecified (Table 1) (8, 10–18, 21–50). Additionally, 27 women (19%) had progression of VTE despite adequate anticoagulant treatment (13–15, 24, 25, 29, 32, 35, 36, 48, 50–59), and 9 women (5%) had a proximal DVT and a contraindication for anticoagulant treatment due to significant risk of bleeding (8, 13, 18, 24, 25, 48, 51). Deep-vein thrombosis and heparin induced thrombocytopenia occurred in 5 women (3%) (24, 25, 35, 51, 60). In more than half of the women (107/199, 54%), an inferior vena cava filter was inserted in the third trimester of pregnancy (Table 1).

**Table 1:** Characteristics, indications and timing of insertion of inferior vena cava filters during pregnancy.

**Indications for filter**	**1st trimester** ** (*n =* 29)**	**2nd trimester** ** (*n =* 26)**	**3rd trimester** ** (*n =* 107)**	**Trimester not reported** ** (*n =* 37)**	**TOTAL** ** (*n =* 199)**
**Filter type** ***, n***					
Permanent					45
Retrievable					154
**Position of filter** ***, n***					
Suprarenal					96
Infrarenal					42
Not reported					61
**Filter shape** ***, n***					
Umbrella-shaped: *Greenfield, Günther Tulip, ALN, Tempofilter, Celect, Recovery*					102
Spindle-shaped: *TrapEase, OptEase, Neuhaus Protect, Antheor*,					38
Free struts and barbs: *Bird's Nest*					2
Undetermined					57
**Indications for filter** ***, n***					
Proximal DVT	7	10	43	30	90
Pulmonary embolism with/without concomitant DVT	7	3	7	0	17
Venous thromboembolism (location not reported)	6	6	27	0	39
Distal DVT	0	0	1	0	1
DVT (location not reported)	2	0	10	0	12
Pulmonary embolism or extensions of DVT despite anticoagulant treatment for initial DVT	4	5	15	3	27
Proximal DVT and contraindication for anticoagulant treatment because of ongoing bleeding or risk of bleeding	3	2	1	2	8
DVT and heparin induced thrombocytopenia	0	0	3	2	5
**Total**	29	26	107	37	199

DVT, deep-vein thrombosis.

Proximal DVT was defined as a thrombus involving one or more of the following veins: the popliteal, femoral, iliac veins, the inferior vena cava.

Distal DVT was defined as infrapopliteal DVT without extension to proximal veins (popliteal vein or above) or pulmonary embolism.

### Obstetric outcomes

Obstetric outcomes were reported in 162 cases: 73 women (46%) had a vaginal delivery and 85 women (52%) had a caesarean section. Four women (2%) had a medically indicated termination of pregnancy. No fetal deaths were recorded. Two neonates (1%) suffered from mild respiratory distress (51), but data concerning the fetal outcomes were often lacking.

### Filter complications

Individual data on follow-up of inferior vena cava filters after insertion in pregnant women were reported for 177 women: at least one complication of the inferior vena cava filter was reported in 33 women (19%). Filter complications are summarized in Table 2. Immediate complications (within 24 h of filter insertion) occurred in three women (2%) and long-term complications (days to months after filter insertion) occurred in 30 women (17%). Two maternal deaths (1%) were reported: one woman had a fatal air embolism during the insertion of a Kimray-Greenfield filter (53), the other woman with an in-filter thrombosis died as a consequence of catastrophic antiphospholipid syndrome (13). The most frequently reported complication was in-filter thrombosis. Some authors reported in-filter thrombosis as a consequence of extended proximal DVT (13, 54, 58, 61), while others described captured thrombi as a successful filter function or as a consequence of discontinuation of anticoagulant therapy (12, 17, 20, 41). Of the 14 in-filter thromboses (8%), concomitant symptomatic pulmonary embolism was reported in one woman (54). These in-filter thromboses or captured thrombi were observed in almost all types of retrievable filters (Celect, Neuhaus Protect, Antheor, OptEase) and in one case with a permanent filter (Greenfield). Filter complications occurred in 21% (20 of 96 women) of suprarenal positioned and in 24% (10 of 42 women) of infrarenal positioned inferior vena cava filters. Overall the complications occurred with all types of filters. Therefore, it is not possible to clearly establish a link between a type of filter and a type of complication. Of note, the level of DVT that justified the need for filter placement in these women was femoral in four women (12, 58), iliofemoral in four women (12, 17, 20, 61), and not specified in six women (13, 18, 41, 54). Among the women with in-filter thrombosis, time since filter insertion was 5 days or less for three women (21%) (12, 61) and 7 days or more for 11 women (79%).

**Table 2:** Immediate and long-term complications of inferior vena cava filters inserted during pregnancy.

**Type**	**Position of filter**	**Name and Type of filter**	**Filter shape**	**Number of patients**	**Outcome**	**Reference**
**Immediate complication (≤24 h after insertion)**						
Air embolism	1 unknown	Greenfield (permanent)	Umbrella	1	Maternal death	(53)
Misplacement of filter (iliac vein)	1 infrarenal	Celect or ALN or Günther Tulip (retrievable)	Umbrella	1	Unsuccessful filter retrieval	(13)
Allergic reaction	1 suprarenal	Neuhaus Protect (retrievable)	Spindle	1	Fully recovered	(11)
**Long-term complication (days to months after insertion)**						
Filter tilt	3 infrarenal 2 suprarenal 1 suprarenal	3 Günther Tulip (retrievable) 2 OptEase (retrievable) 1 Recovery (retrievable)	Umbrella Spindle Umbrella	6	Unsuccessful filter retrieval: 3 Successful filter retrieval: 3	(37, 39, 57)
Filter migration	1 infrarenal 1 suprarenal 1 suprarenal 2 suprarenal	Recovery (retrievable) Neuhaus Protect (retrievable) Tempofilter (retrievable) ALN (retrievable)	Umbrella Spindle Umbrella Umbrella	5 ^¥^	Unsuccessful filter retrieval: 2 Successful filter retrieval: 3	(11, 23, 32, 35, 57)
Filter thrombosis including thrombus captured in filter	1 Unknown 2 infrarenal 1 infrarenal (death) + 1 suprarenal 4 suprarenal 1 suprarenal 1 suprarenal 2 suprarenal 1 suprarenal	Neuhaus Protect (retrievable) Neuhaus Protect (retrievable) Celect or ALN or Günther Tulip (retrievable) Unknown Neuhaus Protect (retrievable) Antheor (retrievable) OptEase (retrievable) Greenfield (permanent)	Spindle Spindle Umbrella Spindle Spindle Umbrella Umbrella	14 ^§^	Maternal death: 1^*^ Pulmonary embolism: 1	(12, 13, 17, 18, 20, 41, 54, 58, 61)
Filter fracture	1 Unknown 1 infrarenal 1 suprarenal	Neuhaus Protect (retrievable) Recovery (retrievable) Recovery (retrievable)	Spindle Umbrella Umbrella	3	Unsuccessful retrieval of the filter fragment: 2	(32, 57, 79)
Vena cava perforation	1 infrarenal 1 suprarenal	Greenfield (permanent) Celect (retrievable)	Umbrella Umbrella	2	Unsuccessful filter retrieval: 1 Leading to retroperitoneal haematoma: 1	(8, 80)

¥ Localization of filter migration: right atrium = 2 (one migration to the right atrium resulting in premature ventricular contractions), renal vein = 1, caudal migration = 2.

* Maternal death as a result of catastrophic anti-phospholipid syndrome.

§ captured thrombi > 1 cm was observed in 4 cases.

Other complications of the filter were observed in 19 women and included in a descending order of frequency: tilts (six women, 3%), migrations (five women, 3%), fractures (three women, 2%), vena cava perforation (two women, 1%), misplacement (one woman, < 1%) and allergy (one woman, < 1%). The most important consequence of these complications was the failure of filter retrieval in nine of the women concerned.

### Filter retrieval

In the large majority of women with retrievable filters, the vena cava filter could be retrieved (140/154, 91 %). Data on time to filter retrieval was available for 98 women, in 81 women (83%) the inferior vena cava filter was left *in situ* for a maximum of 30 days and in the remaining 17 women (17%) filters were retrieved after 1 month. For eight of these women (47%), time since filter insertion was more than 90 days with a maximum of 287 days (15). In nine of the 154 women with a retrievable filter (6%) failed attempts of retrieval were reported (8, 13, 35, 36, 39, 57, 59). *Two* of these retrieval failures (22%) occurred after a very long time after insertion (167 and 659 days), the other six attempts (66%) were performed after an *in situ* time varying between 6 and 73 days, and for one woman (11%) data were missing. In five women (5/154, 3%), no attempt of filter retrieval was made and the filter was left *in situ*. The reasons were persistent extensive DVT despite of anticoagulants (62), in-filter thrombosis (41), filter misplacement into external iliac vein (13) or maternal dead (13). Hence, in total 9% of the filters were not retrieved.

## New pregnancy in women with a permanent vena cava filter

In the second part of this review, we aim to evaluate the use, obstetric outcomes, and filter complications of patients who became pregnant with an inferior vena cava filter already *in situ* prior to conception.

## Literature search—methods

Similar to the first part of the review, a literature search was conducted. However, a review on this exact same subject has been recently performed and published by one of the authors of this review (63). In that publication a comprehensive search of the English language literature was conducted in MEDLINE, Embase, and abstracts of conferences between 1970 and August 2020 (63). For the current review, we have repeated the same search for the period from August 2020 to May 2022 (Figure 1). No restrictions with regard to study design nor geographic location were applied. Articles were included if they reported data on pregnancies after insertion of an inferior vena cava filter that was left *in situ*. Information about filter indication, route and timing of filter insertion, filter complications, indwelling time, and maternal and fetal outcomes was collected. All reference lists of included manuscripts were manually searched to identify related articles that were not yet identified.

## Results

The extended literature search yielded seven new articles based on titles and abstracts, and only one article was included after full text screening. The reasons for exclusion of the six other manuscripts were: review articles (*n* = 2), filter insertion during pregnancy or postpartum (*n* = 3), and no inferior vena cava filter inserted (*n* = 1) (Figure 1). The included study was a case series of Taiwanese patients with inferior vena cava thrombosis (64). This case series included one 46-year old woman who was pregnant and had an unretrieved inferior vena cava filter *in situ*. However, other than the inferior vena cava thrombosis, no details or outcomes of interest were reported. The recently published review (63) revealed one cohort study (13), two case series (36, 48) and two case reports (65, 66). Additionally, the review also reported data from its own cohort. In total, data on 21 pregnancies in 14 women were available.

### Filter insertion

Among 14 women, six women (43%) had a permanent vena cava filter (3 Bird's Nest, 1 Greenfield, 2 TrapEase) inserted, six women (43%) had a retrievable inferior vena cava filter (2 Günther Tulip, 2 OptEase, 2 undetermined retrievable filter) inserted, and for two women (14%) the filter type was unknown. Of the women with a retrievable filter, retrieval attempts failed in five women (83%) and in one woman (17%) no attempts were made. The filter position was infrarenal in six women (43%) and was not reported for the other eight (57%) women. Indication for an inferior vena cava filter was pre-pulmonary endarterectomy because of chronic thrombo-embolic pulmonary hypertension in two women (14%) (63), pulmonary embolism or recurrent VTE and contraindication for anticoagulant therapy due to surgery or bleeding in three women (21%) (36, 63, 65), DVT or pulmonary embolism during pregnancy in four women (29%) (13, 36, 63), recurrent VTE despite anticoagulant therapy in two women (14%), and VTE outside of pregnancy in two women (14%) (48, 66). The indication was unknown in one woman (64). Time between filter insertion and pregnancy ranged from < 1–8 years.

### Obstetric outcomes

Obstetric outcomes were reported for 17 pregnancies: 15 pregnancies (87%) ended in life-births, one pregnancy (7%) ended in miscarriage before the 10th weeks of gestation (63), and one pregnancy (7%) ended in an emergency cesarean section at 24 weeks of gestation (65). The later was the result of a filter complication described below. The fetus died shortly after birth.

### Filter complications

Filter complications were reported for 16 pregnancies and summarized in Table 3. In 14 pregnancies (88%) no complications occurred, but follow-up and imaging of the filter was poorly performed. One pregnancy (6%) was complicated by uterine trauma and major intraperitoneal hemorrhage caused by perforation of the vena cava wall and uterus by the inferior vena cava filter's barbs and struts (65). In this case, the infrarenally positioned TrapEase filter was already known to have perforated the inferior vena cava wall prior to pregnancy, but the woman had been asymptomatic up until the uterine laceration occurred (65). Other filter complications were reported by one other study (64), information was limited to the occurrence of inferior vena cava filter thrombosis. It was not reported whether this was caused by an in-filter thrombosis.

**Table 3:** Complications of inferior vena cava filters in women with new pregnancy with inferior vena cava filter *in situ*.

**Complication**	**Number of patients**	**Outcome**	**Reference**
Perforation of vena cava wall and uterus by filter barbs and struts of TrapEase filter in infrarenal position	1	Uterine trauma Massive intra-abdominal bleeding Emergency cesarean section Fetal death	(65)
Inferior vena cava thrombosis	1	Unknown	(64)

## Discussion

Our literatures searches compiled data on 199 women who had an inferior vena cava filter inserted during pregnancy, and data on 21 pregnancies that occurred in 14 women who had an inferior vena cava filter *in situ* prior to conception. In women who had a filter inserted during pregnancy, 77% had a retrievable filter and in more than half of these women the filter was inserted in the third trimester of pregnancy. At least one complication was reported in 19% of women, most women had in-filter thrombosis. Two women died after filter insertion, however for one of them it was unclear whether this was a direct complication of the filter insertion. Retrieval failure was reported in 6%. These numbers are comparable to the nonpregnant population. In women who became pregnant with a filter *in situ*, complications were poorly evaluated but one filter complication resulting in major hemorrhage and fetal death was reported.

Although VTE risk increases up to 7–10-fold during pregnancy compared to age-matched controls, the overall incidence remains low (around 1–2 per 1,000 pregnancies) (67). Consequently, it is not surprising that the number of pregnant women who had an inferior vena cava filter inserted for an acute VTE reported in the English literature was low: only 199 cases have been reported since 1970 and no randomized-controlled trials on the safety and efficacy of inferior vena cava filters in pregnancy have been conducted. Moreover, the very low number of women who became pregnant with an inferior vena cava filter *in situ* was also expected. In the recent American Society of Hematology (ASH) guideline on venous thromboembolism management in pregnant women, the question whether to insert a vena cava filter for the treatment of acute VTE in the third trimester of pregnancy has not been addressed (68). The older American College of Chest Physicians (ACCP) guideline discusses the use of vena cava filters which was restricted to women with very high risk of recurrence, such as women with proven DVT and recurrent pulmonary embolism despite anticoagulant treatment (69).

From the data provided in this review, we can conclude that most women who had an inferior vena cava filter inserted during pregnancy did not meet these indications and should not have had a filter inserted. At most, only 4 % of the women had an absolute contraindication for anticoagulant therapy and failure of anticoagulant treatment was the indication for filter insertion in 14% of pregnant women. The occurrence of VTE was the most frequently reported reason for filter insertion, while patients were not at very high risk of recurrence. This might be based on the fear of a pulmonary embolism occurrence or recurrence related to the temporary withdrawal of anticoagulant treatment peripartum. Higher VTE incidence during the third trimester of pregnancy and in the early postpartum period is well reported (70, 71), but the risk of thrombosis extension or new pulmonary embolism some hours after anticoagulation withdrawal is poorly evaluated in the literature. There is one retrospective study reporting 344 nonpregnant patients with VTE who had a vena cava filter inserted and received no anticoagulants. In 42% of patients there was a contraindication for anticoagulants because of a significant risk of bleeding. These patients were matched using propensity scores with 344 other patients treated with only anticoagulants without having a vena cava filter inserted. After 30 days of treatment, the risk-adjusted pulmonary embolism related mortality rate was lower for filter insertion compared to no filter insertion (1.7 vs. 4.9%; *p* = 0.03), but the risk-adjusted recurrent VTE rates were higher for filter insertion compared to no filter insertion (6.1 vs. 0.6%; p < 0.001) (72). The authors concluded that despite an increased risk of VTE events, including in-filter thrombosis, filter insertion did not allow for a large pulmonary embolism to occur (73).

The most frequently reported filter complication was in-filter thrombosis. This is a well-known complication of vena cava filters and usually occurs at long-term use (>30 days) (7, 74). Early in-filter thrombosis has also been described as a captured large thrombus that can appear only a very few days after its insertion (11, 12, 17). These findings argue for optimal peripartum management and require a multidisciplinary approach: the window without anticoagulant therapy should kept as short as possible and both induction of labor and bridging with unfractionated heparin should be considered. Furthermore, anticoagulant therapy should be resumed as soon as possible after delivery and filter retrieval should be planned. The incidence of other filter complications is lower and similar to incidence rates of the nonpregnant population. Some authors suspected that during the second stage of labor and delivery intra-abdominal pressure could cause tilt, fracture and migration of the filter (20, 75). Due to the low number of patients in our review, we were unable to statistically compare such complications for patients with vaginal delivery compared to patient who had a caesarean section. Finally, the rate of filter complications in our review might be overestimated because of selection and publication biases.

The failure rate of filter retrieval is low (6 %) and comparable to the one in nonpregnant population (76, 77). However, in 3% no filter retrieval was attempted. When the filter remains *in situ*, women will be exposed to complications described by Decousus in a nonpregnant cohort with a follow-up period of 8 years (78), these include DVT recurrence and in-filter thrombosis.

In conclusion, only in pregnant women with clear indication such as acute proximal DVT shortly prior to delivery and contraindication for anticoagulant therapy, or progression of DVT despite adequate anticoagulant therapy, should inferior vena filters be considered. When inserted, retrieval should be planned as soon as possible and temporary filters are to be preferred over permanent filters. This would help to avoid long-term complications in young women who might be planning future pregnancies.

## Author contributions

IB and AB performed the literature searches, interpreted extracted data, and wrote the first draft of the manuscript. BT critically reviewed and revised the manuscript. The final version of the manuscript was approved by all authors.

## Conflict of interest

The authors declare that the research was conducted in the absence of any commercial or financial relationships that could be construed as a potential conflict of interest.

## Publisher's note

All claims expressed in this article are solely those of the authors and do not necessarily represent those of their affiliated organizations, or those of the publisher, the editors and the reviewers. Any product that may be evaluated in this article, or claim that may be made by its manufacturer, is not guaranteed or endorsed by the publisher.
